# Gutta-Percha in Crown Setting

**Published:** 1903-04-15

**Authors:** Howard Williamson


### GUTTA-PERCHA IN CROWN SETTING.

BY HOWARD WILLIAMSON.

By the aid of a copper crown setter a perfect joint is made between the Logan crown and root with a gutta-percha washer. The gutta-percha is made pliable and held so by heat passing from the crown setter to crown and platinum pin until a perfectly burnished joint is made.

This has advantage over cement of not being acted on by secretions of the mouth.

The crown can then be cemented permanently after the root has been treated and the apical end filled with Oxpara,

